# 大网膜胸腔内移植覆盖支气管残端治疗肺切除术后支气管胸膜瘘（附6例报道）

**DOI:** 10.3779/j.issn.1009-3419.2018.03.26

**Published:** 2018-03-20

**Authors:** 晓樽 杨, 晓军 杨, 天鹏 谢, 彬 胡, 强 李

**Affiliations:** 1 610000 成都，成都医学院 Chengdu Medical College, Chengdu 610000, China; 2 610041 成都，电子科技大学医学院附属肿瘤医院胸外科中心 Department of Thoracic Surgery, Sichuan Cancer Hospital and Institute, Sichuan Cancer Center, School of Medicine, University of Electronic Science and Technology of China, Chengdu 610041, China

**Keywords:** 肺切除术, 支气管胸膜瘘, 补救性手术, 大网膜胸腔内移植, Lobectomy, Bronchopleural Fistula, Remedial Operation, Omentum Transplantation in Thorax

## Abstract

**背景与目的:**

支气管胸膜瘘（bronchopleural fistula, BPF）是胸外科肺切除术后常见并发症，临床治疗复杂且效果不佳。对于肺切除术后支气管胸膜瘘的处理一直困扰着胸外科医生。总结我院胸外科中心予大网膜胸腔移植覆盖支气管残端治疗肺切除术后出现BPF的临床资料，分析出现BPF的原因，总结外科治疗的方法，探讨其可行性、安全性及小样本的成功率。

**方法:**

2016年8月-2018年2月，我中心对接受肺切除术后发生BPF的患者6例，进行再次开胸补救性手术、大网膜胸腔内移植覆盖支气管残端治疗。2例首次手术行肺叶切除（分别为右肺上叶及中下叶切除，再次手术均行患侧残肺切除，直线切割器缝合主支气管），4例首次手术行全肺切除（左右各2例）。术中予4-0微荞线修补主支气管残端后于心膈角处打开膈肌将大网膜移植入胸腔内后覆盖支气管残端。S术后予生理盐水浸泡胸腔。回顾分析上述6例患者的临床资料，总结该术式治疗肺切除术后BPF的临床效果。

**结果:**

6例均为男性，中位年龄66岁（61岁-73岁）；术后发生BPF中位时间为术后25天（10天-45天）。再次手术中位时间为110 min（80 min-150 min），术中中位出血量450 mL（200 mL-1, 000 mL），再次手术后住院时间中位天数14天（12天-17天）。6例患者术后均恢复良好痊愈出院，支气管残端闭合良好，成功率为100%。随访1个月-18个月各病例均未再出现BPF相关并发症。

**结论:**

肺切除术后发生BPF，如患者全身情况尚可耐受手术，应尽早行补救性手术，带蒂大网膜瓣容易获取，胸腔内移植覆盖支气管残端疗效确切可靠，值得临床推广应用。

## Abstract (en)

**Background and objective:**

Bronchial pleural fistula (BPF) is a common complication after thoracic surgery for lung resection. Clinical treatment is complex and the effect is poor. The treatment of BPF after lung resection has plagued thoracic surgeons. We reviewed retrospectively the clinical and follow-up data of 6 patients in our hospital who underwent the omentum transplantation in thorax to cover bronchial stump as treatment of BPF after pulmonary resection to analyze why BPF occurs and describe this treatment method. We intend to discuss and evaluate the feasibility, safety and small sample success rate ofthis treatment method.

**Methods:**

During August 2016 to February 2018, six patients in our hospital underwent remedial open thoracotomy and omentum transplantation in pleura space to cover bronchial stump as treatment of bronchopleural fistula after pulmonary resection. Four patients had undergone a prior pneumonectomy and two patients had undergone a prior lobectomy (the residual lungs were resected with the main bronchus cut by endoscopic stapler during the reoperation). The bronchial stumps were sutured by 4-0 string with needle and covered by omentums, which were transplanted in pleura space from the cardiophrenic angle. Postoperatively, the pleura space was irrigated and drained. Summarize the clinical effect and technique learning points.

**Results:**

The patients were all males, aged 61 to 73 years (median age: 66). BPF occurred from postoperative day 10 to 45 (median postoperative day 25). The reoperation was finished in 80 mins-150 mins (median 110 mins). Total blood loss was 200 mL-1, 000 mL (median 450 mL). These patients were discharged on postoperative day 12-17 (median 14 days), and there was no more complications associated with bronchopleural fistula. All six patients' bronchial stumps were well closed (100%) and have recovered well during the follow-up period, which lasted 1 month-18 months.

**Conclusions:**

Remedial operation should be performed as soon as possible when BPF after pulmonary resection diagnosed. Excellent prognoses can be achieved by omentum which is easy to get transplanted in thorax to cover bronchial stump as treatment in patients with BPF after pulmonary resection those who can tolerate reoperation.

支气管胸膜瘘（bronchopleural fistula, BPF）在胸外科肺切除术后严重并发症中较为常见，死亡率高^[1]^。肺切除术后出现BPF常伴胸腔感染或继发脓胸，常规胸腔引流及内镜治疗临床效果较外科治疗不佳，存在复发可能^[2]^。我院采用再次开胸手术行支气管瘘修补并予大网膜胸腔移植覆盖支气管残端治疗肺切除术后BPF 6例，临床效果良好，现报告如下。

## 1 资料与方法

### 1.1 临床资料

2016年8月-2018年2月，我科采用大网膜胸腔移植覆盖支气管残端治疗肺切除术后BPF 6例，均为男性，中位年龄66岁，年龄分别为73岁、70岁、62岁、61岁、65岁、67岁；右全肺切除2例，左全肺切除2例，右肺上叶切除1例，右肺中下叶切除1例；病种为肺癌患者5例，肺结核瘤患者1例。6例患者确诊BPF中位时间为术后25天（10天-45天）；5例行胸部增强计算机断层扫描（computed tomography, CT）及纤维支气管镜确诊，1例患者术后出现持续刺激性咳嗽，咯出胸液样痰。6例患者确诊BPF后均二次开胸行支气管瘘修补并予大网膜胸腔移植覆盖支气管残端治疗肺切除术后支气管胸膜瘘（2例肺叶切除患者二次开胸均行残肺切除）。

### 1.2 手术方法

确诊BPF应立即行患侧胸腔闭式引流并嘱患侧卧位以免胸液污染健肺。尽快完成常规术前准备后，予患者全身麻醉双腔气管插管，2例首次开胸手术经原切口进胸，4例首次胸腔镜肺切除术（video-assisted thoracic surgery, VATS）后经第5肋间后外侧切口进胸。首先彻底清除胸腔内积液及支气管旁胶冻样组织，4例全肺切除术后患者均充分暴露主支气管残端瘘口并予4-0微荞线间断缝合修补主支气管残端；2例肺叶切除术后患者胸腔感染重，残肺复张差，支气管残端瘘口旁炎性坏死组织与残肺支气管及血管分界不清，难以充分暴露支气管瘘口，评估后行残肺切除，予直线切割缝合器结扎离断主支气管并予4-0带针缝合线间断缝合加固主支气管残端（图 1B）。生理盐水浸泡胸腔予患侧支气管通气确保无漏气。然后于心膈角处打开膈肌（图 1A）游离并牵拉部分大网膜组织移植入胸腔（图 1C），包埋并覆盖于支气管残端后缝合膈肌（图 1D）。检查无活动性出血后予碘伏、生理盐水反复浸泡、冲洗胸腔，于腋中线第7肋间安置胸腔引流管后关胸。术后予敏感抗生素抗感染并予营养支持治疗。

**1:**
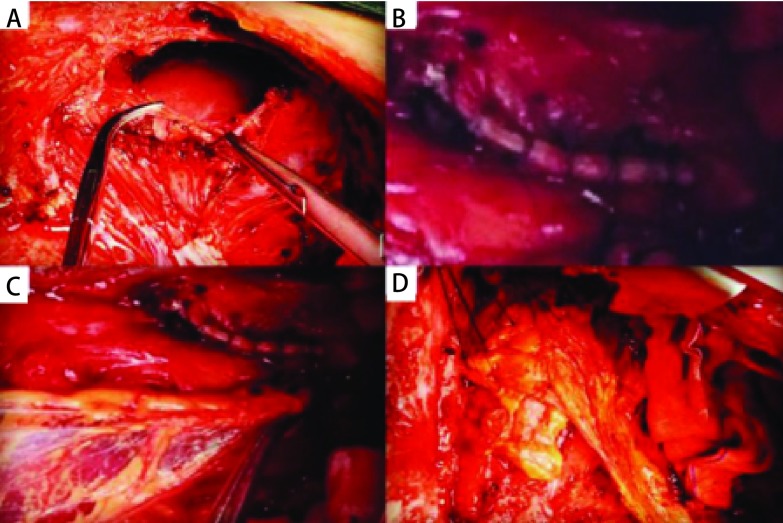
修补主支气管残端后将大网膜移植入胸腔内后覆盖支气管残端。A：于心膈角处打开膈肌；B：予4-0带针缝合线间断缝合加固主支气管残端；C：游离并牵拉部分大网膜组织移植入胸腔；D：大网膜包埋并覆盖支气管残端。 Repair the main bronchus stump andtransplant the omentum in pleura space to cover bronchial stump. A: Diaphragm was opened at the cardiophrenic angle; B: Bronchial stump was sutured by 4-0 string with needle; C: The omentum was transplanted in pleura space; D: The bronchial stump was covered by omentum.

## 2 结果

6例患者再次手术中位时间为110 min（80 min-150 min），术中中位出血量450 mL（200 mL-1, 000 mL），再次手术后住院时间中位天数14天（12天-17天）。

6例患者术后均恢复良好痊愈出院，支气管残端闭合良好，成功率为100%（图 2）。随访1个月-18个月未出现相关并发症。

**2:**
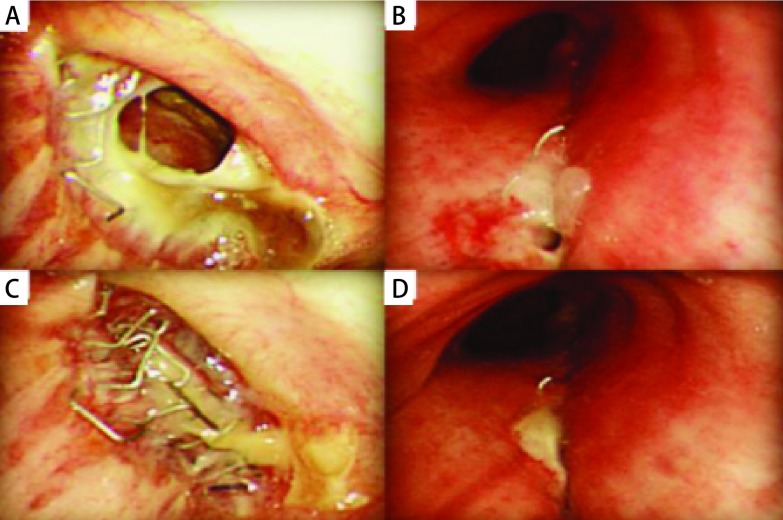
纤支镜示出现不同程度的BPF术后支气管残端闭合良好。A、B：纤支镜下可见不同程度的BPF；C、D：术后复查纤支镜见支气管残端闭合良好。 Varying degrees of BPF were showed in bronchoscope after surgery the bronchial stumps were well closed. A, B: Varying degrees of BPF were showed in bronchoscope; C, D: Bronchial stumps were well closed. BPF: bronchopleural fistula.

## 3 讨论

近年来，随着外科技术与材料的进步以及术后抗生素的应用的规范化，肺切除术后支气管胸膜瘘发生率较前有所降低，国外文献报道肺切除术后发生率为0.80%-12.5%。全肺切除术后BPF发生率较肺叶切除术后BPF发生率高，右全肺术后更甚^[3-5]^。目前我中心肺切除术后支气管胸膜瘘的发生率已控制在1%以下的水平。然而肺切除术后BPF一旦发生，必然合并脓胸，并发严重感染时常引起呼吸衰竭及多器官功能衰竭严重危及患者生命。

查阅文献并回顾我中心临床资料探讨肺切除术后发生BPF原因：①术前呼吸功能异常伴慢性阻塞性肺疾病，长期使用皮质醇激素，低蛋白血症及患者合并糖尿病与肺切除术后BPF的发生具有显著相关性^[2]^；②术中淋巴结清扫、支气管残端过分剥离影响支气管血供，肿瘤残留易导致术后BPF，也有文献报道胸腔粘连是术后发生BPF的独立相关因素^[6]^；③术后胸腔感染、应用机械通气使气道长期处于高压状态亦是BPF发生的危险因素。

肺切除术后BPF的治疗需个体化^[7]^。危重患者不可耐受手术可采用纤支镜下支气管瘘口激光烧灼、蛋白胶等封堵剂封堵，但对于8 mm以上BPF，内镜治疗成功率较低，复发率高^[8, 9]^。对于全身情况尚可耐受手术的肺切除术后BPF患者，应尽早再次开胸行补救性手术，过去行胸膜余肺切除或胸廓成形术，该术式创伤大，易造成血管意外损伤及大出血；术后胸腔内积液积血容易导致BPF复发。近年来，根据支气管残端情况直接或修整残端后再缝合支气管残端并予自体组织覆盖加固支气管残端，可有效治疗肺切除术后BPF。相关文献报道^[7, 10, 11]^，带蒂肋间肌瓣或纵隔、心包胸膜加固支气管残端治疗支气管残端瘘有效，但此术式操作难度大，技术要求高，且对于肺切除术后BPF合并脓胸，上述组织覆盖患者支气管残端局部感染风险可能增加。

大网膜具有分泌、吸收、保护、抗炎和再生特性，且血运丰富，是治疗肺切除术后BPF的理想材料^[12-14]^。本组6例肺切除术后BPF患者中4例为全肺切除，2例为肺叶切除（残肺部分实变再次手术时行残肺切除），行再次开胸手术时发现患者胸腔感染严重合并脓胸，首先彻底清除胸腔内积液及支气管旁胶冻样组织。4例全肺切除患者充分暴露主支气管残端瘘口并予4-0带针缝合线间断缝合修补主支气管残端，2例需行残肺切除患者予直线切割缝合器结扎离断主支气管并予4-0带针缝合线间断缝合加固主支气管残端。查无漏气后于心膈角处打开膈肌游离并牵拉部分大网膜组织移植入胸腔，包埋并覆盖于支气管残端后缝合膈肌。操作简单，创伤小，出血少，且6例患者均恢复良好痊愈出院，支气管残端闭合良好，成功率为100%。目前虽然为小样本资料，但初期临床应用的如此良好的结果，我们仍然可以较为乐观地认为，予修补主支气管残端后将大网膜移植入胸腔内后覆盖支气管残端符合肺切除术后发生BPF充分引流、关闭瘘口消灭脓腔的治疗原则，临床效果好。本方法存在的缺点为：手术范围扩大至腹腔，易导致腹腔感染；同时更全面客观的结论得出需要研究和总结更多大样本和多中心的临床资料。

总之，对于肺切除术后发生支气管胸膜瘘，如患者全身情况尚可耐受手术，应尽早行再次开胸补救性手术，予修补主支气管残端后将大网膜移植入胸腔内后覆盖支气管残治愈成功率高，创伤小，值得临床推广。
